# Impact of a Mobile Health System on the Suppression of *Schistosoma haematobium* in Chad

**DOI:** 10.4269/ajtmh.20-1151

**Published:** 2021-08-16

**Authors:** Didier Lalaye, Mirjam E. de Bruijn, Tom P. V. M. de Jong

**Affiliations:** ^1^ Julius Global Health Center, University Medical Center Utrecht, Utrecht, The Netherlands;; ^2^ African Studies Centre, Leiden University, The Netherlands;; ^3^ University Children’s Hospitals UMC Utrecht, Utrecht, The Netherlands;; ^4^ Amsterdam UMC, Amsterdam, The Netherlands

## Abstract

This study determined the contribution of a mobile health (M-health) system to the treatment of *Schistosoma haematobium* in a region of Chad where *S. haematobium* is endemic. M-health involves the use of a mobile phone for health care. The study compared the prevalence of schistosomiasis in an area with an M-health system, newly installed in 2014, with an area without an adequate health infrastructure. Data were gathered after the M-health system had been running for 3 years. We took urine samples from children age 1 to 15 years, for a total of 200 children in a village in the M-health area and 200 in a village in a non-M-health area. Urine was checked for urinary schistosomiasis by using dipsticks for microhematuria and, in cases of positive dipstick results, microscopy was used to detect eggs. Comparison between the areas allowed us to assess the effectiveness of the installed M-health system after 3 years of operation. Based on dipstick outcomes, the non-M-health area had an infection rate of 51.5% compared with 29% in the M-health area. Microscopy results in non-M-health and M-health were 27.5% and 21%, respectively. The dipstick result difference between M-health and non-M-health areas was statistically significant. Dipsticks were more reliable than microscopy for the detection of schistosomiasis, especially in areas without qualified personnel. Based on these results, M-health proved its ability to reduce the infection rate of urogenital schistosomiasis, and the implementation of M-health shows great promise in areas where this disease is endemic and where no mass drug administration is provided.

## INTRODUCTION

Bilharziasis, or schistosomiasis, is a parasitic disease caused by trematodes, which are hematophagous flatworms of separate genders. Adult flatworms live in the circulatory system of mammals and evolve during the larval stage in freshwater mollusks.^1^ In cases of *Schistosoma haematobium*, the female worm produces eggs that penetrate the urinary bladder, producing symptoms of a lower urinary tract disease. Changes in the bladder wall may lead to malignancies of the bladder at a later age. Clinical signs may vary from hematuria with few extra complaints, to severe forms of irritative bladder complaints and obstruction of the upper urinary tract caused by fibrosis of the ureters. The most commonly used tests to diagnose the disease are detection of hematuria by urine test strips and direct detection of eggs using a microscope for a centrifuged or filtered urine sample.^2^

Schistosomiasis is one of the most prevalent parasitic infections worldwide and has a significant impact on the physical condition of a population. It influences socioeconomic conditions and has severe public health consequences.^3^ Although the distribution of schistosomiasis has changed during the past 50 years—as a result of the success of several control projects, among other reasons—the number of people infected or at risk for infection has not declined.^4^ It is estimated that 250 million people are infected, of whom 120 million are symptomatic, 20 million have severe disease, and approximately 700 million people worldwide are at risk for infection.^5^

The health system in Chad is poorly developed, especially in remote rural areas. Health services there are quasi-absent and, if they do exist, the equipment for diagnosis and treatment of diseases is poor, and the majority of health staff are unqualified. The region has one district hospital in Torrock and eight health centers, only two of which (Gouin and Balani) had laboratory facilities before the start of this study. The national health programs in Chad for selected diseases are based in regional hospitals, which are difficult for poor people to reach because of long travel distances. *Schistosoma haematobium* is not part of a national health program. A number of basic diseases, such as schistosomiasis, have relatively easy diagnostics and treatment. In contrast to many other countries in West and Central Africa, no specific investigations have ever been made in Chad on the prevalence of *S. haematobium* and related kidney diseases, with the exception of our earlier study.^6^

Mobile health (M-health) systems are becoming increasingly important in developing countries. These systems involve the use of portable electronic devices to facilitate health care. To find a solution for schistosomiasis in Chad, we developed an M-health system for the control and treatment of the disease, using a short message service (SMS) relay system via mobile phone. This system to overcome the absence of health care/services was installed in Torrock, a village in the center of a large area with endemic schistosomiasis. The system has been functioning since March 2015.

The project began with an awareness-raising campaign launched in 2014. The Torrock laboratory was equipped with a microscope and centrifuge for egg detection, and local personnel were trained. In March 2015, an SMS relay system was implemented in which children’s urine samples were acquired at their home and the samples were examined at the newly installed laboratory in the village. In the case of a positive diagnosis, medication for treatment (praziquantel) was forwarded to the child’s home. The chain starts when a village representative sends, at the request of parents, an SMS to a local health worker who coordinates the control and treatment. The local health worker collects a urine sample, sends it to the laboratory where it is examined, and, if the sample proves positive, a doctor prescribes the dosage of medication. The medication is then provided by the pharmacist and taken to the family of the sick child.^6^ *All* communications among health worker, doctor, and pharmacist are by SMS.

At the start of the project, we determined the prevalence of *S. haematobium* in the region (Mayo Dallah). To do this, we took 1,875 samples in the region between March 2015 and March 2016. The samples revealed 467 positive cases (a prevalence of 24.9%) based on microscopy of centrifuged urine for eggs.^6^

Our current study was conducted from July to August 2017 in two villages: Torrock, with its M-health service in operation since March 2015, and Rong, where there was no M-health service. The two villages are in the same district and are approximately 15 km apart. The objective of the study was to determine the effect the M-health system had on the infection rate in the population 2 years after the introduction of the system by comparing the prevalence of schistosomiasis in the two areas.

## MATERIALS AND METHODS

### Sampling.

As mentioned, the investigation was performed at two collection points, one in Torrock and one in Rong. A sample of urine was taken from children 1 to 15 years old. All samples were acquired between July 15 and August 30, 2017, and a minimum sample size was set at 200 children age 1 to 15 years, regardless of gender, selected randomly from each village. A local health worker enrolled the children in a register with a code assigned to each child. A questionnaire was completed for all children to determine whether they had symptoms of infection with *S. haematobium*. Urine samples were taken between 9 am and 1 pm, and all children were weighed. The urine, when positive for hematuria in a dipstick test, was collected in plastic jars and transported within 4 hours to the health center laboratory for microscopic analysis for the presence of eggs. Only samples with positive results from the hematuria dipstick test were centrifuged and examined via microscopy. All positive cases tested by dipstick were treated with free praziquantel, provided by the regional hospital.

### Area of study.

The villages of Torrock and Rong each have a population of approximately 4,000 inhabitants. Exposure of the population to infected water is via rice cultivation, one of the main agricultural activities in the region, and via the herding of cattle. The area has no electricity.

### Target group.

Inclusion was possible for all children age 1 to15 years, regardless of gender, residing in the two villages.

### Data analysis.

Data were collected in an Excel spreadsheet (Microsoft^®^ Excel^®^ 2016, Microsoft^®^ Excel^®^ 2019 MSO, 32 bits, Seattle, WA) for each village separately and were analyzed using SPSS 25 software (IBM^®^ SPSS^®^ Statistics, New York, NY), with 5% as the threshold of significance. Given the objective of the study, we wished to determine whether there was a difference in the proportion of *S. haematobium* incidence between the test populations in Rong and in Torrock, and, if so, whether this difference was statistically significant.

### Assumption of the study and statistics.

We took p1 and p2 to be the proportions of children infected with *S. haematobium* in Torrock and Rong, respectively. Our H1 hypothesis was that p1 ≠ p2; that is, there was a significant difference in the prevalence of *S. haematobium* between the village with an M-health system and the village without one. A left-tailed test that we called Z was used. The criterion for accepting one of the hypotheses was as follows: for 5% as the threshold of significance, the confidence interval was between –1.96 and +1.96, which meant our H1 hypothesis would be rejected if Z fell within the confidence interval; if Z did not, H1 would be accepted.

### Ethics.

Before commencing the study, an application for authorization was sent to the sub-prefect of Torrock and to the various administrative, religious, and educational authorities of the villages. An authorization was granted by the health delegate of Mayo-Dallah for the establishment of three laboratories in the region for the analysis of urine. These authorization measures functioned as an institutional review board because an ethical committee does not exist in the country. Before urine sampling and examination, parents of the children provided oral consent after being informed. Written consent was not possible because the vast majority of the population cannot read or write. Data were collected anonymously.

### Patient and public involvement.

The local population was not involved in the design of the study. The study was announced publicly, before its start, in churches, mosques, schools, and public areas in the villages.

## RESULTS

The overall results for the entire sample size of 400 individuals are presented and split between the village with and the village without M-health. Table 1 shows the gender distribution in both areas; Table 2 shows the distribution of children according to microscopy examination and by village; Table 3 shows the distribution of children according to dipstick test and by village; and Table 4 shows the relationship between results for hematuria observed by dipstick test and microscopy examination.

**Table 1:** Gender distribution in the mobile health and non-mobile health villages

Village	Female	Male	Total
Mobile health, *n *(%)	98 (49)	102 (51)	200 (100)
Non-mobile health, *n *(%)	94 (47)	106 (53)	200 (100)
Total, *n *(%)	192 (48)	208 (52)	400 (100)

**Table 2:** Distribution of children according to microscopy examination and by village

Village	Microscopy results		Total
	Negative	Positive	
Mobile health, *n* (%)	158 (79.0)	42 (21.0)	200 (100.0)
Non-mobile health, *n* (%)	145 (72.5)	55 (27.5)	200 (100.0)
Total, *n* (%)	303 (75.8)	97 (24.3)	400 (100.0)

**Table 3:** Distribution of children according to dipstick test and by village

Village	Dipstick results		Total
	Negative	Positive	
Mobile health, *n* (%)	142 (71.0)	58 (29.0)	200 (100.0)
Non-mobile health, *n *(%)	99 (49.5)	101 (50.5)	200 (100.0)
Total, *N*	241	159	400

**Table 4:** Relationship between results for hematuria observed by dipstick test and microscopy examination

Dipstick test results	Microscopy results		Total
	Negative	Positive	
Dipstick positive results			
* n*	62	97	159
* *% of total	39.0	610	100.0

**Figure 1.**
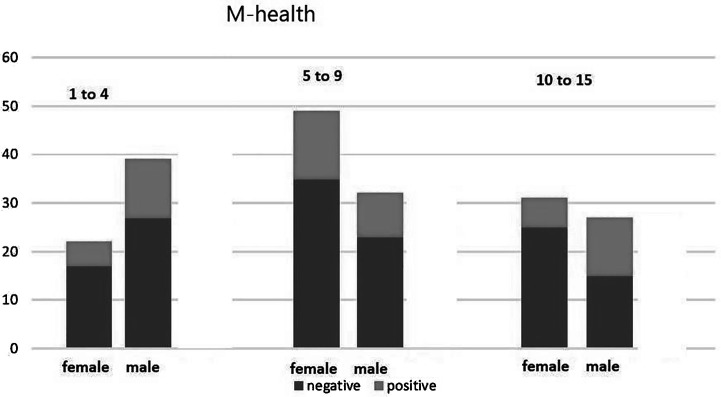
Distribution by gender and age in mobile health (M-health) area based on dipstick results.

**Figure 2.**
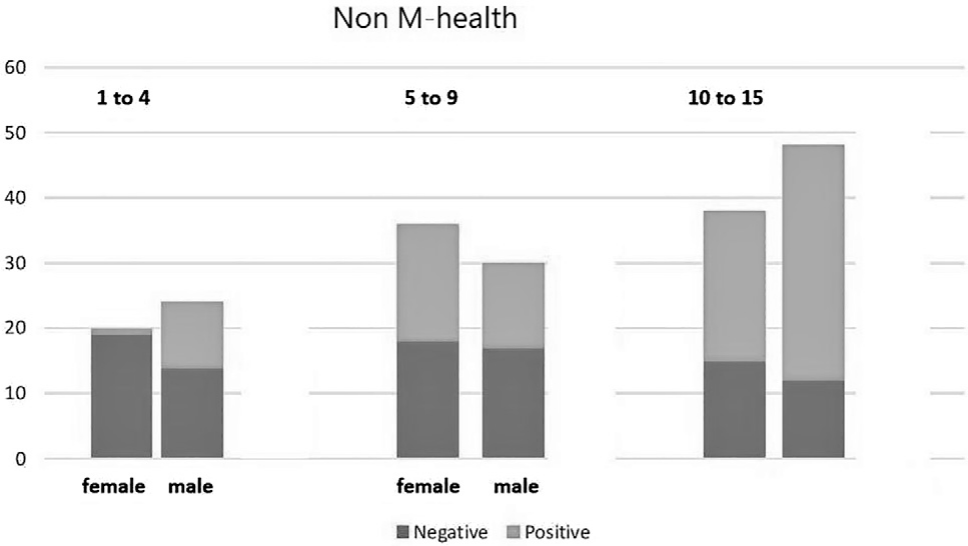
Distribution by gender and age in non-mobile health (M-health) areas based on dipstick results.

Figures 1 and 2 present the numbers of positive and negative cases in three age groups. Although some differences in numbers per age group existed between the M-health and non-M-health areas, this did not seem to influence the outcome.

Looking at the microscopy results, the prevalence of *S. haematobium* in the M-health village was 21% and, in the non-M-health village, was 27.5%. The Z value of –1.52 was in the confidence interval. Thus, by using microscopy only, we found no significant difference in the prevalence of *S. haematobium* between the villages, despite the introduction of M-health in Torrock.

Looking at the dipsticks results, the prevalence of *S. haematobium* in the M-health area was 29% and in the non-M-health area was 50.5%. The Z value of –4.48 was not in the confidence interval, so we accepted our H1 hypothesis; there was a significant difference in terms of *S. haematobium* prevalence between the village with M-health and the one without M-health.

Of 159 cases exhibiting a positive urinary dipstick result, only 61% could be confirmed by finding eggs with microscopy. Of the 62 children showing hematuria with a dipstick test but no positive eggs by microscopy, 43 reported positive lower urinary tract symptoms by questionnaire. Among the 97 positive cases, both by test strip and microscopy, 47 had no symptoms reported by questionnaire.

## DISCUSSION

This study examined a total of 400 children, equally divided between two different areas: an M-health zone and a non-M-health zone. Gender and age distribution in both areas was comparable. Looking at positive hematuria results from the dipstick test, a significant difference was found in positive cases between the M-health and non-M-health villages, indicating that M-health is a system that provides a promising means to control *S. haematobium*

Of 159 positive cases detected by hematuria strip, eggs were found by microscopy in only 61% of cases (97 children). Of the 62 children with hematuria but without positive eggs found with microscopy, 43 reported positive symptoms by questionnaire. For field studies in remote endemic areas without infrastructure, the dipstick test is considered reliable with small numbers of false-positive and false-negative outcomes.^2^

**Figure 3.**
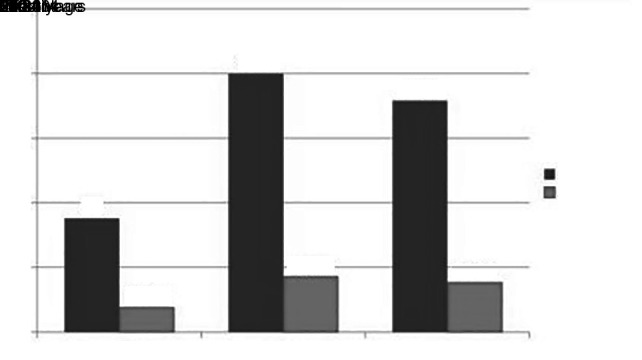
Microscopy-based study of the prevalence of *Schistosoma haematobium* in 1,875 children as a zero measure before the current study was performed.^6^

The high rate of negative microscopy may have been caused by a lack of experience in the health-care workers involved in the study. This conclusion is underscored by the comparison of Figures 2 and 3. Figure 3 is from a study conducted as a zero measure with microscopy only (no dipstick was used for hematuria, unlike in our current study). One would expect this to match Figure 2 in the non-M-health area. The striking difference can be explained if, in the study shown in Figure 3, 40% of cases was missed because of a failure to find eggs with microscopy.^6^

According to King et al.,^7^ (2013), the results of dipstick and egg detection depend on many factors. They noted that the presence of eggs in hematuria is lower among treated populations (72%), and population subgroups having lower-intensity infection (65%). This finding is in accordance with our findings that eggs were found in 67% of the cases of hematuria, although we did not find an important difference between M-health and non-M-health villages in the percentage of positive microscopy results. King et al.^7^ also found that when the insensitivity of egg-count testing was considered (and diagnosis inferred instead from combined hematuria and egg-count findings), overall dipstick sensitivity and specificity was 82% and 97%, respectively, with significantly better sensitivity (92%) in settings of high prevalence of schistosomiasis.

We can conclude that 1) dipstick testing is very useful and more reliable than microscopy for the detection of urinary schistosomiasis, and 2) that the absence of eggs does not mean an absence of infection. Thus, bias can be minimized by using dipsticks instead of microscopy for the detection of schistosomiasis. In addition, in cases when only a microscope test is used for the diagnostics of schistosomiasis, it is likely to result in undertreatment of an important part of the population. Ochodo et al.^2^ (2015) estimated that in a group of 1,000 people, of whom 410 have urinary schistosomiasis detected in microscopy testing, using the hematuria strip would mis-classify 77 uninfected individuals as infected; these individuals would thus receive unnecessary treatment. At the same time, microscopy would wrongly classify 102 infected people as uninfected, who would thus not receive any treatment.^2^

The prevalence of *S. *haematobium in the non-M-health village was comparable to the prevalence in the M-health village as measured during the baseline study, when the samples were checked with microscopic egg detection. In our study of the effect of the M-health system on the prevalence of *S. haematobium*, the outcome was disappointing when we considered the microscopy results. Compared with the study that we conducted during the introduction of the M-health system in Torrock, the prevalence did not decline as much as one might have expected. This may have been a result of the fact that the urine samples of the 1,875 children were examined under microscopy only, which, in retrospect, underestimated the true prevalence.^6^

On the other hand, the results were encouraging when we considered the dipstick test. Shehata et al.^8^ found, after a single mass drug administration, a reduction in prevalence of *S. haematobium* from 28.6% at baseline to 20.3% after 1 year, rising to 38% after 4 years. Comparing the effect of the M-health system with the effect of such a single mass administration of praziquantel, the effect of M-health is promising.

The proliferation and adoption of mobile phone technologies within the global south have created an opportunity for governments and health-care institutions to establish contact with millions of people who otherwise would be out of reach. Many studies have identified mobile phones as the main devices used in health-care systems, apart from other devices such as smartphones and the Internet.^9^ Our study of the treatment of urinary schistosomiasis has proved that an M-health system can be inexpensive, rapid to implement, effective, useful in the context of remote areas, and easy to operate.

A limitation of this study is that we have no information on the number of children in the M-health and non-M-health villages that had had earlier treatment with praziquantel. A second limitation is that we have insufficient proof that the prevalence in the two endemic study areas was exactly the same at baseline. Nevertheless, our study demonstrated a successful introduction of M-health to a remote area with a poor health-care infrastructure.

Betjeman et al.^10^ noted that the possible barriers to M-health in sub-Saharan Africa are quite large and are related to strategic leadership, learning environment, capacity for implementation, culture of information use, technology usability, interoperability, privacy and security, sustainable funding, and cost effectiveness. A survey conducted by the WHO in all member states in Africa found that the barriers to investment in and scale-up of M-health programs in the region are primarily the result of operating costs, knowledge, infrastructure, and policy (in increasing order of importance). In our study, we overcame some of these barriers.

The success of mobile digital technologies,^6^ especially mobile phones, in health care is no longer in doubt. A number of current statistical studies illustrate this digital revolution. However, when launching an M-health program, studies need to be conducted on the socioeconomic level of the population; on their beliefs, attitudes, and perceptions regarding diseases in general; and on the existing health-care system in particular.^11^

## CONCLUSION

In our study, 200 children from an M-health village and 200 children from a non-M-health village were screened for urinary schistosomiasis to compare the prevalence of schistosomiasis in the two locations. According to the results from the use of dipsticks, the non-M-health village was positive in more than 50% of cases; for the M-health village, it was positive for 29%. Statistically, there was a significant difference between the two villages. The microscopy results showed 27.5% and 21%, respectively, in the non-M-health and M-health locations, and statistically they did not different significantly from each other. Dipsticks proved to be a more reliable method than microscopy in the detection of *S. haematobium*, and this method is especially useful in remote areas where qualified personnel are lacking.

We conclude that the M-health system that has been introduced in Torrock offers promising results in terms of reducing the burden of schistosomiasis—and possibly reducing the burden of other neglected tropical diseases in the future. Studies over larger areas are required, and we have currently introduced a mobile laboratory in this region of Chad and will report on the results later.
